# Iron Acetate in the Treatment of Pneumonia

**Published:** 1905-06-17

**Authors:** 


### IRON ACETATE IN THE TREATMENT OF PNEUMONIA.

The treatment of pneumonia at the present day is for the most part of the expectant order. It is, therefore, not without interest to note that there are some practitioners who advocate more active measures. Mr. H. J. Robson[1] strongly urges the virtues of acetate of iron obtained by mixing

15 minims of the solution of the perchloride with two drachms of solution of ammonium acetate in half an ounce of chloroform water. This is given to an adult every four hours or, in severe cases, every six hours alternately with 5 minims of solution of strychnine. This scheme, it is urged, is particularly useful in the spreading form of pneumonia following influenza, in catarrhal and lobar pneumonia in debilitated subjects, and in the severe broncho-pneumonia of early life. The crisis of the disease is said to be hastened and such a complication as empyema seldom occurs. Mr. Robson condemns the use of quinine and of antipyretics of the coal-tar series. He uses tepid sponging or the cold pack for high temperatures, but finds that with the above treatment such measures are seldom required. Alcohol he finds unnecessary, except occasionally towards or after the crisis. In regard to the general feeding and care of the patient he lays stress upon the importance of giving abundance of fluid, 3½ pints daily, and he employs, in addition to milk and egg-flip, such foods as liquid peptonoids and plasmon. The former of these he regards as of great value, more especially in young patients. Purgatives, except at the very outset, should be avoided; if there is constipation later it should be treated by enema. Mr. Robson argues that the iron acetate either kills the pneumococcus or neutralises its toxins. He has never found the remedy cause gastric disturbance, and he is not deterred from its use by the existence of a coated tongue or a high-tension pulse.

[1] Brit. Med. Jour., April 15, and May 6, 1905.

